# Supplementary material for: A Prospective Cohort Study Assessing the Relationship between Plasma Levels of Osimertinib and Treatment Efficacy and Safety
Source: Biomedicines. 2023 Sep 10;11(9):2501. doi: 10.3390/biomedicines11092501 (PMC10526408; doi:10.3390/biomedicines11092501)
Supplement: Supplementary file 1 [file biomedicines-11-02501-s001.zip › biomedicines-2579161-supplementary.pptx]

## Slide 1
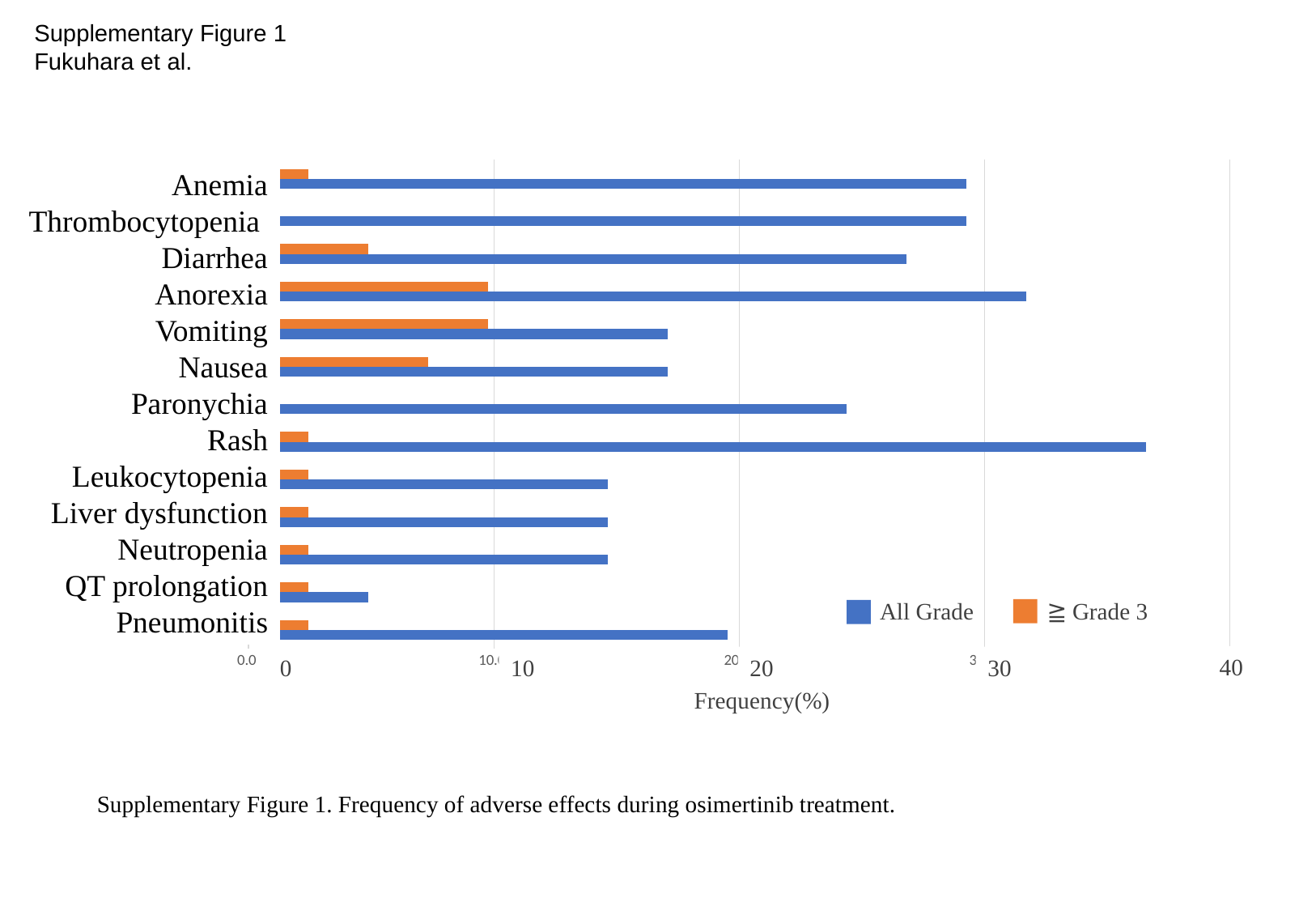

Supplementary Figure 1
Fukuhara et al.
### Chart
| Category | | |
|---|---|---|
| 肺臓炎 | 19.51219512195122 | 2.4390243902439024 |
| QT延長 | 4.878048780487805 | 2.4390243902439024 |
| 好中球減少 | 14.634146341463413 | 2.4390243902439024 |
| 肝障害 | 14.634146341463413 | 2.4390243902439024 |
| 白血球減少 | 14.634146341463413 | 2.4390243902439024 |
| 皮疹 | 36.58536585365854 | 2.4390243902439024 |
| 爪囲炎 | 24.390243902439025 | 0.0 |
| 悪心 | 17.073170731707318 | 7.317073170731707 |
| 嘔吐 | 17.073170731707318 | 9.75609756097561 |
| 食欲不振 | 31.70731707317073 | 9.75609756097561 |
| 下痢 | 26.82926829268293 | 4.878048780487805 |
| 血小板減少 | 29.268292682926827 | 0.0 |
| 貧血 | 29.268292682926827 | 2.4390243902439024 |Anemia
Thrombocytopenia
Diarrhea
Anorexia
Vomiting
Nausea
Paronychia
Rash
Leukocytopenia
Liver dysfunction
Neutropenia
QT prolongation
Pneumonitis
All Grade
≧ Grade 3
40
0
10
20
30
Frequency(%)
Supplementary Figure 1. Frequency of adverse effects during osimertinib treatment.

## Slide 2
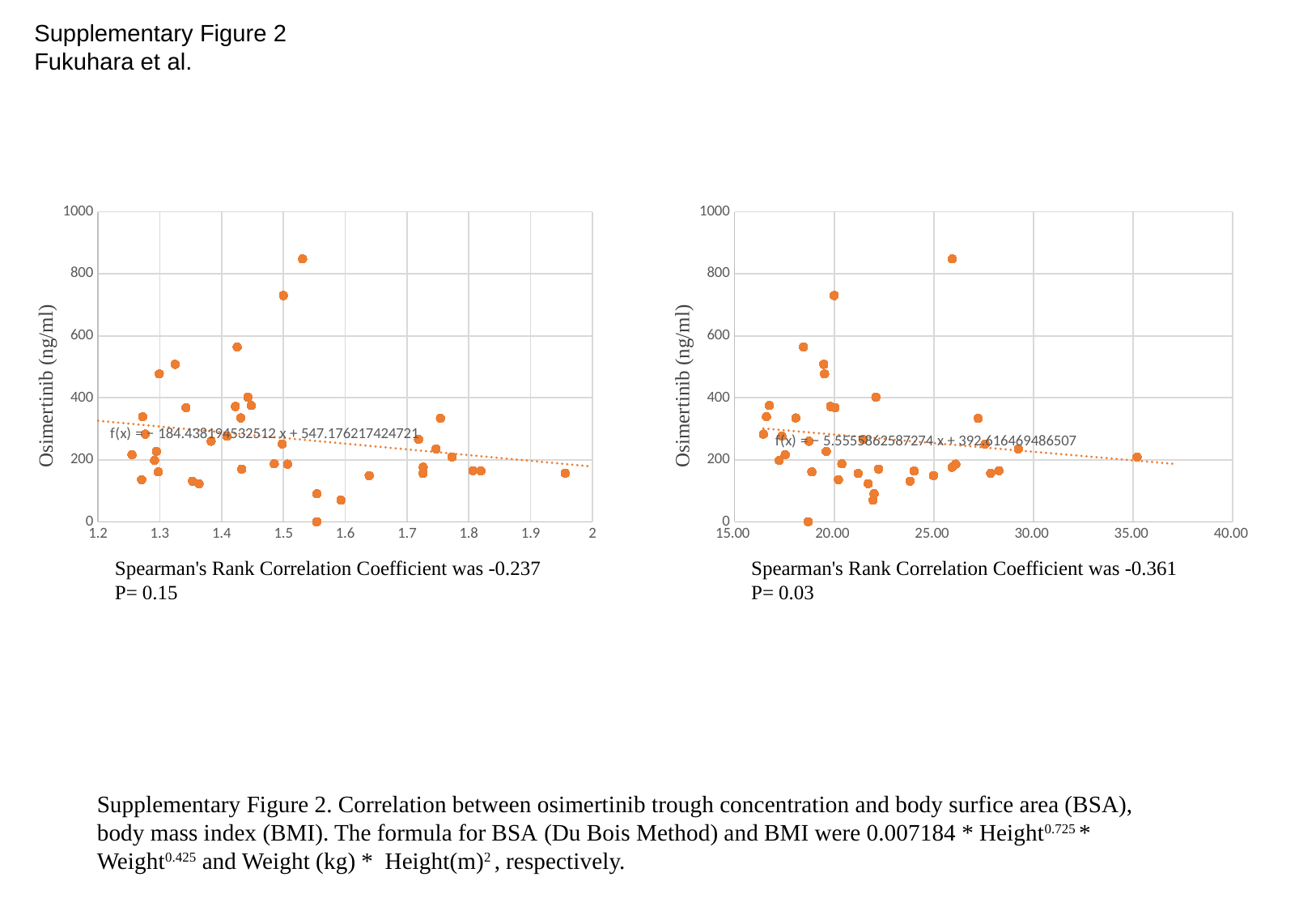

Supplementary Figure 2
Fukuhara et al.
### Chart
| Category | 登録番号 |
|---|---|
### Chart
| Category | 登録番号 |
|---|---|Osimertinib (ng/ml)
Osimertinib (ng/ml)
Spearman's Rank Correlation Coefficient was -0.237
P= 0.15
Spearman's Rank Correlation Coefficient was -0.361
P= 0.03
Supplementary Figure 2. Correlation between osimertinib trough concentration and body surfice area (BSA), body mass index (BMI). The formula for BSA (Du Bois Method) and BMI were 0.007184 * Height0.725 * Weight0.425 and Weight (kg) * Height(m)2 , respectively.

## Slide 3
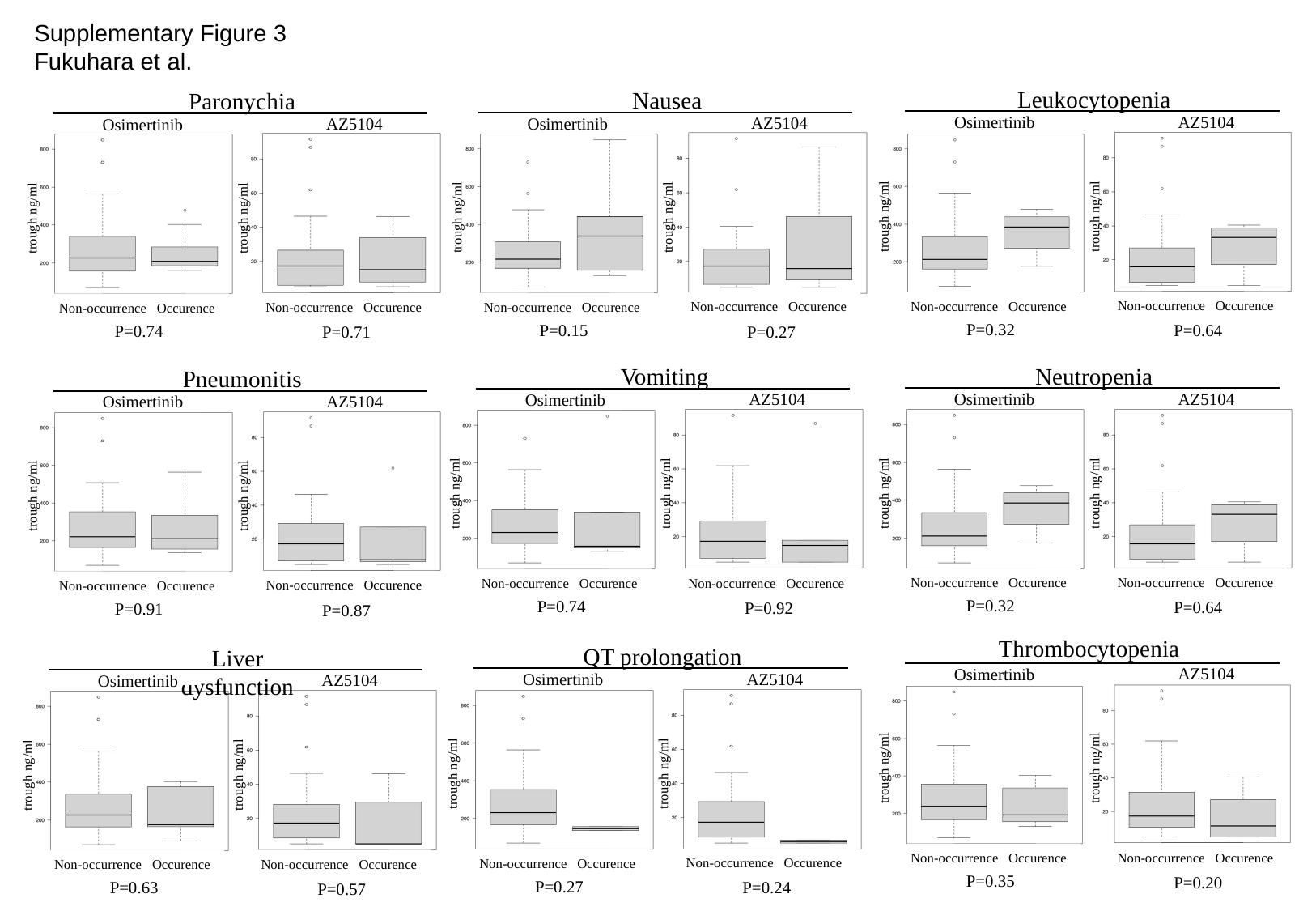

Supplementary Figure 3
Fukuhara et al.
Leukocytopenia
AZ5104
Osimertinib
trough ng/ml
trough ng/ml
Non-occurrence Occurence
Non-occurrence Occurence
P=0.32
P=0.64
Nausea
AZ5104
Osimertinib
trough ng/ml
trough ng/ml
Non-occurrence Occurence
Non-occurrence Occurence
P=0.15
P=0.27
Paronychia
AZ5104
Osimertinib
trough ng/ml
trough ng/ml
Non-occurrence Occurence
Non-occurrence Occurence
P=0.74
P=0.71
Neutropenia
AZ5104
Osimertinib
trough ng/ml
trough ng/ml
Non-occurrence Occurence
Non-occurrence Occurence
P=0.32
P=0.64
Vomiting
Pneumonitis
AZ5104
Osimertinib
trough ng/ml
trough ng/ml
Non-occurrence Occurence
Non-occurrence Occurence
P=0.91
P=0.87
AZ5104
Osimertinib
trough ng/ml
trough ng/ml
Non-occurrence Occurence
Non-occurrence Occurence
P=0.74
P=0.92
Thrombocytopenia
AZ5104
Osimertinib
trough ng/ml
trough ng/ml
Non-occurrence Occurence
Non-occurrence Occurence
P=0.35
P=0.20
QT prolongation
AZ5104
Osimertinib
trough ng/ml
trough ng/ml
Non-occurrence Occurence
Non-occurrence Occurence
P=0.27
P=0.24
Liver dysfunction
AZ5104
Osimertinib
trough ng/ml
trough ng/ml
Non-occurrence Occurence
Non-occurrence Occurence
P=0.63
P=0.57

## Slide 4
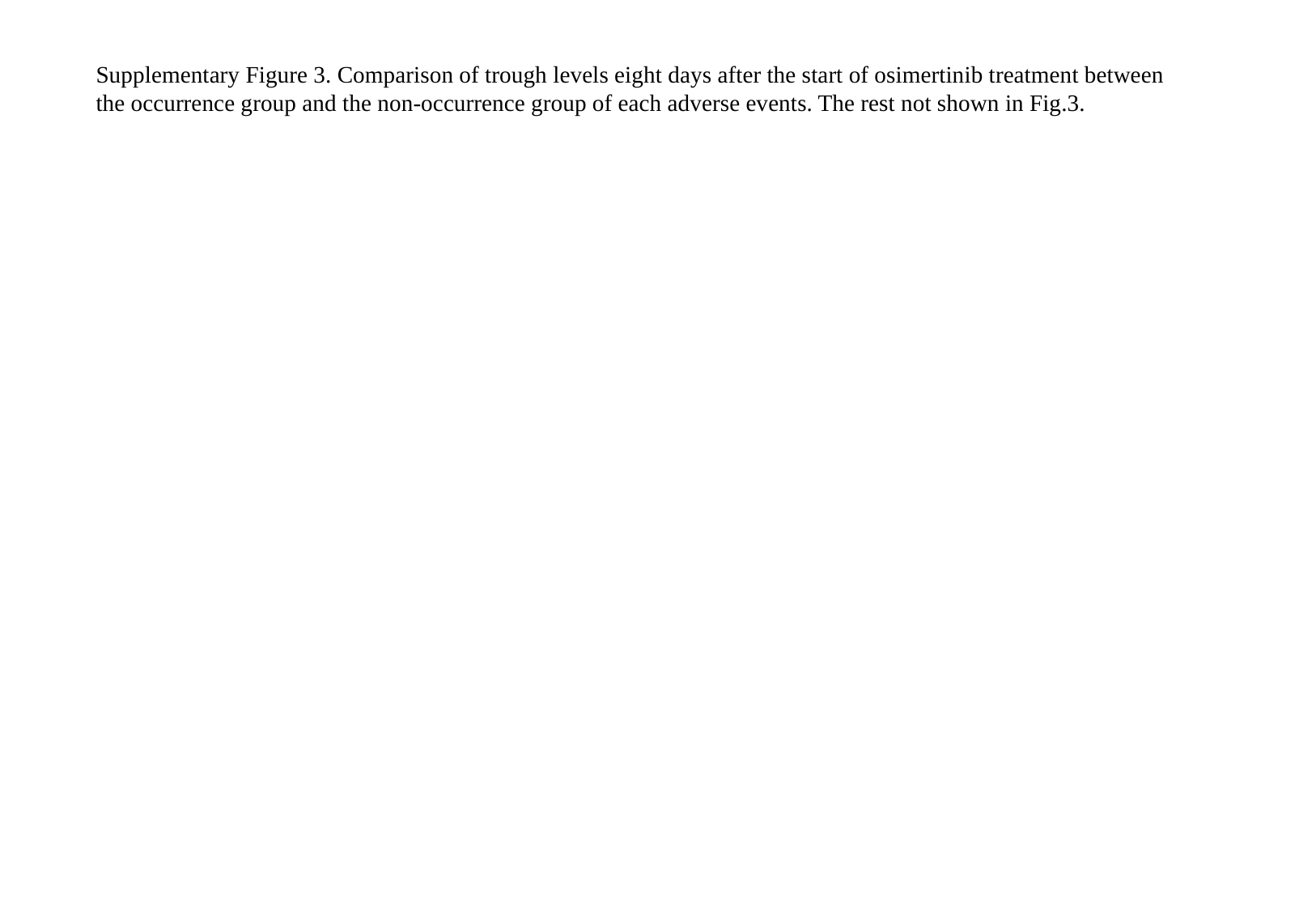

Supplementary Figure 3. Comparison of trough levels eight days after the start of osimertinib treatment between the occurrence group and the non-occurrence group of each adverse events. The rest not shown in Fig.3.

## Slide 5
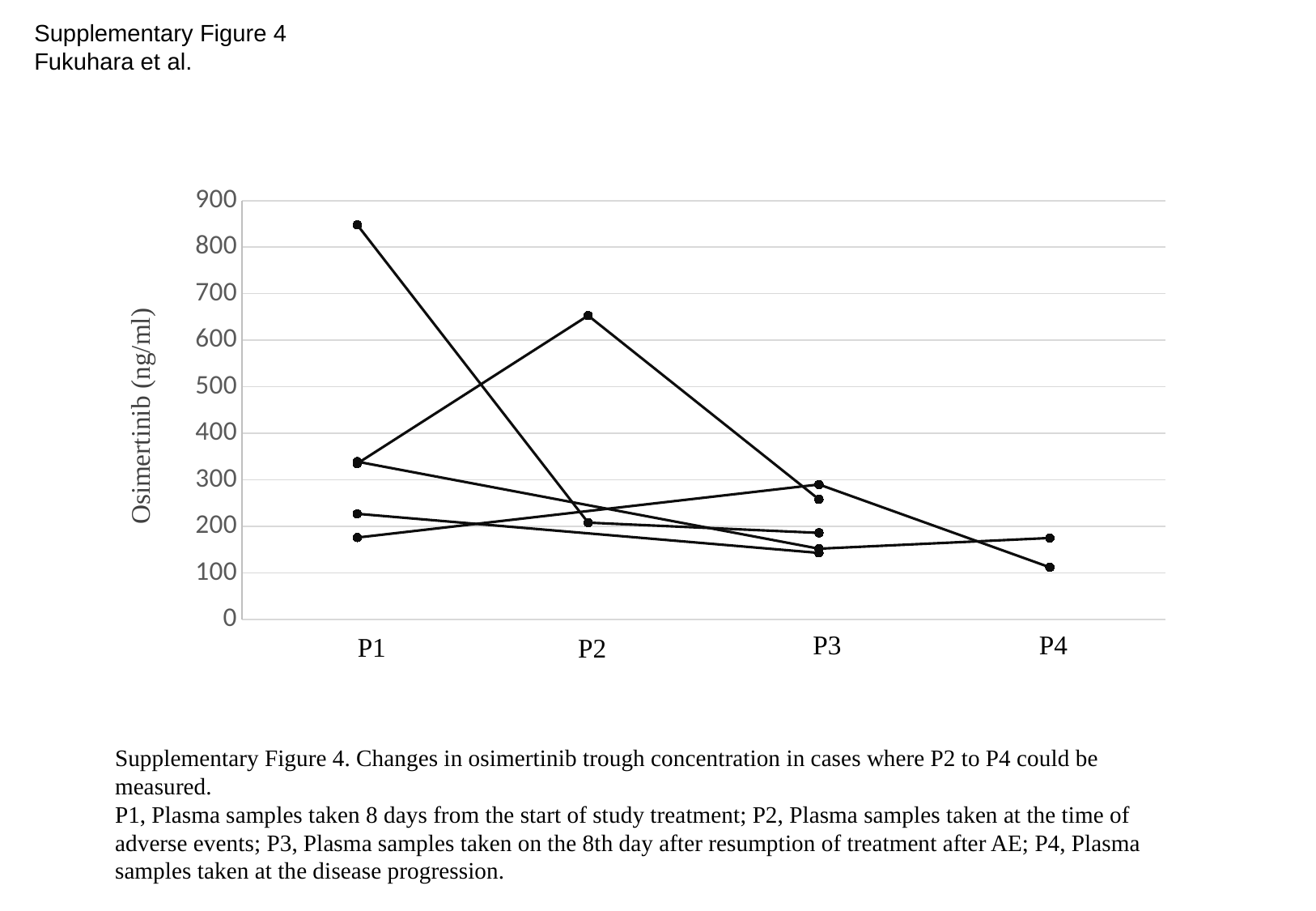

Supplementary Figure 4
Fukuhara et al.
### Chart
| Category | OC-19 | OC-21 | OC-33 | OC-34 | OC-41 |
|---|---|---|---|---|---|Osimertinib (ng/ml)
P3
P4
P1
P2
Supplementary Figure 4. Changes in osimertinib trough concentration in cases where P2 to P4 could be measured.
P1, Plasma samples taken 8 days from the start of study treatment; P2, Plasma samples taken at the time of adverse events; P3, Plasma samples taken on the 8th day after resumption of treatment after AE; P4, Plasma samples taken at the disease progression.
